# Batch variability and anti-inflammatory effects of iPSC-derived mesenchymal stromal cell extracellular vesicles in osteoarthritis *in vitro* model

**DOI:** 10.3389/fbioe.2025.1536843

**Published:** 2025-04-02

**Authors:** Maria Elisabetta Federica Palamà, Cansu Gorgun, Matteo Rovere, Georgina M. Shaw, Daniele Reverberi, Matteo Formica, Emanuele Quarto, Frank Barry, Mary Murphy, Chiara Gentili

**Affiliations:** ^1^ Department of Experimental Medicine (DIMES), University of Genoa, Genoa, Italy; ^2^ Regenerative Medicine Institute (REMEDI), University of Galway (UoG), Galway, Ireland; ^3^ UOC Research-Scientific Direction, Istituto di Scientifico Ricovero e Cura a Carattere, Ospedale Policlinico San Martino, Genoa, Italy; ^4^ UOC Clinica Ortopedica, Istituto di Scientifico Ricovero e Cura a Carattere, Ospedale Policlinico San Martino, Genoa, Italy; ^5^ UOC Oncologia Cellulare, Istituto di Scientifico Ricovero e Cura a Carattere, Ospedale Policlinico San Martino, Genoa, Italy

**Keywords:** extracellular vesicle (EV), mesenchymal stromal cells, induced pluripotent derived mesenchymal stromal cells, osteoarthritis, inflammation

## Abstract

Mesenchymal stromal cell-derived extracellular vesicles (MSC-EVs) hold promise as a cell-free therapy for osteoarthritis (OA), due to their immunomodulatory and anti-inflammatory properties. However, the need for large-scale expansion to obtain MSC-EVs for clinical use can lead to senescence-related changes and loss of stem-like properties. In this scenario, induced pluripotent stem cell (iPSC)-derived MSCs (iMSCs) offer the unique opportunity to address obstacles associated with traditional MSC-based therapies. This study used a xeno-free (XFS) medium for long-term expansion of both MSCs and iMSCs, and their EVs comparison. Characterization of both cells and EVs was conducted across different passages, and the anti-inflammatory potential of EVs and iEVs was assessed using an *in vitro* model of osteoarthritis. Long-term expansion of MSCs resulted in cellular senescence and a reduction in trilineage differentiation capacity by passage five, accompanied by diminished anti-inflammatory properties of EVs. On the other hand, iMSCs exhibited batch-to-batch variability in differentiation and EV biological properties. However, the effects of iMSC-EVs were prolonged compared to MSC-EVs, providing a wider window of activity for therapeutic purposes. Despite this, the variability among iMSC batches poses challenges for their reliability in OA treatment. Further work is needed to overcome these limitations for clinical application.

## 1 Introduction

Osteoarthritis (OA) is the most common chronic joint disorder affecting elderly populations. Deterioration of cartilage, ligaments and menisci, osteophyte outgrowth, and low-grade synovitis are the major pathological phenomena characterizing OA progression (Kraus et al., 2015). Although the exact pathogenesis of OA remains unclear, the establishment of an inflammatory milieu has been associated with the progression of OA and most age-related diseases (Coryell et al., 2021). Among the therapeutic strategies being investigated for OA treatment, the use of mesenchymal stromal cells (MSC)-derived extracellular vesicles (EVs) holds great promise, offering disease-modifying benefits, through immunomodulating and anti-inflammatory properties (Li et al., 2019; Palamà et al., 2023). MSC-derived EVs carry several advantages over the use of MSCs for clinical intent, providing comparable biological activity, without the potential risks of a cell-based therapy (Bjørge et al., 2018).

However, donor-dependent variability, low cell isolation yield and inadequate scalability are substantial challenges often associated with primary donor-derived MSCs (Martin et al., 2019). Extensive *in vitro* culture expansion of MSCs is required to produce large numbers of therapeutic cells and EV doses. This may lead to replicative senescence of the cells, reduced multipotency and metabolic changes, which heavily affect the restorative properties of MSC products (Stenderup et al., 2003; Xu et al., 2017).

To address these issues, recent studies have focused on the generation of MSCs from induced pluripotent stem cells (iPSC), as a novel cell source for tissue regeneration (Zhang et al., 2021). iPSCs offer enormous advantages for tissue regeneration, due to their high proliferative and differentiation potentials (Diederichs and Tuan, 2014). Several protocols for generating iPSC-derived MSC-like cells (iMSCs) have already been described (Abdal et al., 2019). These cells closely resemble their primary counterparts in terms of morphology, immunophenotype, and three-lineage differentiation potential, enabling the possibility for a prolonged expansion without senescence-related modifications (Sabapathy and Kumar, 2016).

Furthermore, the therapeutic effects of iMSC-derived EVs have also been explored in many diseases such as cardiovascular disease, musculoskeletal pathology and in the modulation of allogeneic immune responses, where different experimental settings, such as depleted-FBS or human platelet lysate, were employed for the cultivation of iMSCs (Levy et al., 2023; Hong et al., 2024; Meng and Guo, 2023; Tertel et al., 2023).

However, optimal culture conditions should be identified to obtain high-grade cell products to be translate to the clinic, animal-free media represent an appropriate alternative for cell growth for clinical application. We previously demonstrated the beneficial effects of a patented xeno-free supplement (XFS), through enhancement of MSC anti-inflammatory properties, through the secretion of both chondroprotective molecules (Palamà et al., 2020) and EV-encapsulated therapeutic miRNA (Palamà et al., 2023).

In this study, we therefore compared and characterized MSCs and iMSCs during long-term growth in the XFS-containing medium, assessing cell proliferation, senescence, phenotype, and three-lineage differentiation including chondrogenesis. Furthermore, we describe the characterization and *in vitro* biological validation of both MSC- and iMSC-derived EVs (iEVs) to ensure not only sustainable, reproducible, and validated cell sources but also standardized iEV products for use as a future therapeutic agent for OA patient cohorts.

## 2 Material and methods

### 2.1 Generation of multiple iMSC lines from a single iPSC line

The iPSC line ChiPS 22 (Takara Bio, Sweden, European Stem Cell Registry no. CEBi001-A) was plated and maintained in DEF-CS medium (Takara Bio, Sweden) for 2 days. The medium was changed to STEMdiff mesoderm induction medium (Stem cell Technologies, Canada) and maintained with daily medium changes for 4 days. The culture medium was then switched to a MSC culture medium consisting of Alpha-MEM-GlutaMAX (GiBCo, Waltham, MA, United States), supplemented with a mixture of 100 U/mL penicillin and 100 mg/mL streptomycin (GiBCo, Waltham, MA, United States), along with the Xeno-free Purstem supplement (XFS) (patent No. PCT/EP2015/053223) and the culture maintained for a further 2 days. At this point the cultures were re-plated in the MSC medium onto fibronectin coated cell culture plastic. To obtain iMSC cultures, 2-3 subsequent passages were necessary, without the requirement for fibronectin coating. At passage 4-5 cells have a fibroblastic-like appearance and were further characterized for MSC characteristics by surface marker expression analysis and trilineage differentiation. Three different batches of iMSCs were generated from the same iPSC line and they will be named as SD1, SD2 and SD3.

### 2.2 Primary cell culture

Human bone marrow stromal cells (MSCs) were isolated from heparinised bone marrow aspirates taken from the iliac crest of healthy volunteers with informed consent (average age 23 years, 4 males and 2 females). All procedures for the collection of bone marrow were approved by the Clinical Research Ethical Committee at University College Hospital, Galway, Ireland (reference: 2/08), and the institutional National University of Ireland Galway Research Ethics Committee (reference: 08/May/14). MSCs were cultured using Alpha-MEM-GlutaMAX (GiBCo, Waltham, MA, United States), supplemented with a mixture of 100 U/mL penicillin and 100 mg/mL streptomycin (GiBCo, Waltham, MA, United States), along with the Xeno-free Purstem supplement (XFS) (Palamà et al., 2023).

Human articular chondrocytes (n = 12, 7 female and 5 male, average age = 63 years) were sourced from the femoral head or femoral condyles and tibial plateau of patients undergoing total hip or knee arthroplasty, adhering to established protocols (Palamà et al., 2023; Palamà et al., 2020). Informed consent was obtained from each patient, and the study received approval from the Ethical Committee of San Martino Hospital (CER Liguria: 372/2019 Genoa, Italy).

### 2.3 Senescence-associated β-galactosidase staining

Senescence-associated β-galactosidase staining was performed to assess senescence levels in MSCs at Passage (P) 2, P5, and P10, and iMSCs at P8, P12, and P16 using a Senescence β-Galactosidase Staining Kit from Cell Signaling Technology, in accordance with the manufacturer’s instructions. Images were captured utilizing a Leica-DM1 microscope, and subsequent analysis was performed using ImageJ software.

### 2.4 MSCs and iMSCs phenotypic characterization by flow cytometry

Phenotypic characterization of both MSCs and iMSCs was performed via flow cytometry by using the MSC Phenotyping Cocktail Kit obtained from Miltenyi Biosciences, Germany, following the manufacturer’s guidelines. Briefly, cells were detached by using Triple Select Enzyme and stained with MSC Phenotyping Cocktail. Data generated from the flow cytometry analysis were processed using FlowJo software and represented as Log fluorescence intensity versus the number of cells. The analysis encompassed three primary cultures of MSCs at P2, P5, and P10, as well as three differentiated iMSCs at P8, P12, and P16.

### 2.5 Growth kinetics

Growth kinetics of both MSCs and iMSCs were analyzed from P2 to P10 and P8 to P16, respectively. For each passage, MSCs were seeded at 5 × 10^3^ cells/cm^2^, while iMSCs were seeded at 7 × 10^3^ cells/cm^2^. Continuous cell culture was performed until cells reached confluency. Subsequently, the cells were harvested using TrypLE Select enzyme (Gibco, Germany) and cell counting was performed utilizing a Neubauer counting chamber. Dead cells were stained with Trypan Blue for accurate counting. The population doubling time was calculated employing a specific formula as *n*° $\;of\;doublings=\log_{2}\frac{counted\;cells}{seeded\;cells}$ .

### 2.6 Trilineage differentiation

MSC trilineage differentiation was assessed at P2, P5, and P10 and iMSCs at P8, P12 and P16. For osteogenesis, cells were seeded in a 24-well plate at a density of 1.5 × 10^4^ cells/cm^2^ and subjected to differentiation in DMEM-HG complete medium supplemented with 100 nM dexamethasone, 10 mM β-glycerophosphate, and 100 μM ascorbic acid (Sigma-Aldrich, St. Louis, MO, United States) for a duration of 21 days. Throughout the culture period, cells were maintained in a humidified incubator at 37°C with 5% CO_2_, and the medium was replaced every 3 days. Upon completion of the differentiation period, cells were fixed with 3.7% paraformaldehyde (PFA) for 15 min and stained with a 2% Alizarin Red S solution (Sigma-Aldrich, St. Louis, MO, United States) for 10 min. The red-stained calcium deposits indicative of osteogenic differentiation was visualized under a microscope, and images were captured using a Leica DM1 microscope.

For adipogenic differentiation, cells were seeded as described above and cultured in complete medium supplemented with 1 μM Dexamethasone, 10 μg/mL Insulin, 200 μM Indomethacin, 500 μM 3-Isobutyl-1-Methyl-Xanthine (Sigma-Aldrich, St. Louis, MO, United States) (induction medium) for 3 days. Medium was then changed, and cells were maintained in complete medium supplemented with 10 μg/mL Insulin (maintenance medium). After three cycles of induction and maintenance, cells were fixed with formal calcium (3.7% paraformaldehyde, 1% CaCl_2_ in H_2_O) for 20 min, washed with 60° isopropyl alcohol for 1 min, and then stained with 0.5% Oil Red O (Sigma-Aldrich, St. Louis, MO, United States) solution in 60° isopropyl alcohol. Intracellular lipid drops stained in red were visualized under the microscope, and images were acquired with a Leica DM1 microscope.

For chondrogenic differentiation, both MSCs and iMSCs at the specified passages were counted, and 250,000 cells per cell type were pelleted in a 15 mL tube. After 24 h, pellet was induced in chondrogenic differentiation medium (DMEM-HG) supplemented with 100 nM dexamethasone, 50 μg/mL ascorbic acid, ITS supplement 1x (Corning, New York, United States), 1 mM sodium pyruvate, and 10 ng/mL TGF-β1 (Peprotech). The medium was changed three times a week for 21 days. Following the differentiation period, the pellets were fixed with 3.7% PFA for 15 min, washed in PBS, and preserved in 70% ethanol for histological analysis post paraffin embedding. Sections of 5 µm thickness were cut on a Leica microtome and dewaxed. The sections were then stained with Alcian blue pH 2.5 staining (BioOptica), following the manufacturer’s instructions, to detect the presence of glycosaminoglycans in the extracellular matrix indicative of chondrogenic differentiation.

### 2.7 Separation and characterization of extracellular vesicles from MSCs and iMSCs

Since both MSCs and iMSCs were continuously cultured in a xeno-free medium, devoid of any serum contamination (Palamà et al., 2020), no cell preconditioning was performed. Cells were maintained at 20% O_2_ and 5% CO_2_ at 37°C and cells were passaged upon reaching confluence. At the end of each 72-h period, conditioned medium (CM) was collected and immediately centrifuged at 300 g for 10 min at 4°C to remove dead cells and debris. Subsequently, the supernatant was centrifuged at 2,000 g for 20 min at 4°C to eliminate apoptotic bodies and then stored at −80°C.

For EV separation, CM from each passage was pooled and subjected to ultracentrifugation. The pooled CM was ultracentrifuged at 10,000 g for 40 min at 4°C and then at 100,000 g for 2 h at 4°C to pellet the EVs. The EV pellets were washed with 1X phosphate-buffered saline (PBS) and finally resuspended in 0.22 μm-filtered PBS before storage at −80°C for further experiments. Ultracentrifugation was performed using a Beckman Coulter ultracentrifuge with swinging bucket rotors type SW41Ti and SW55Ti.

The concentration of membrane-bound proteins on the surface of freshly isolated EVs was measured using the BiCinchoninic Acid (BCA) assay (Thermo Scientific Pierce, Rockford, IL, United States) following the manufacturer’s instructions.

#### 2.7.1 Nanoparticle tracking analysis

EV samples were characterized and quantified using Nanoparticle Tracking Analysis (NTA) performed by Zetaview (Particle Metrix GmbH, Germany). The NTA system was equipped with a sample cell and two lasers (488 nm and 640 nm). Analysis was conducted utilizing Zetaview 8.05.14_SP7 software. Prior to analysis, calibration was performed with 100 nm polystyrene beads. The samples were diluted in 0.22 μm-filtered PBS 1X and injected into the system using a 1 mL syringe. Size distribution analyses were conducted at 11 different positions for each sample (Gorgun et al., 2022).

#### 2.7.2 Western blot

The separated EVs were re-suspended in commercial RIPA buffer (Cell Signalling Tech, United States) containing and protease inhibitor cocktail at 1X concentration (Sigma-Aldrich). Proteins (2 μg) from each sample were loaded onto a 4%–12% NuPAGE Bis-Tris gel sourced from Life Technologies, Carlsbad, CA, United States. Electrophoresis was conducted at 150 V, and the proteins were transferred onto a polyvinylidene fluoride (PVDF) membrane obtained from Millipore, Burlington, MA, United States.

The blot membranes were incubated overnight at 4°C with specific primary antibodies targeting anti-human CD63 (dilution 1:1,000, 10628D, Invitrogen, Waltham, MA, United States), anti-human CD81 (dilution 1:5,000, 555,675, BD Biosciences), anti-human syntenin-1 (dilution 1:1,000, ab133267, Abcam), and anti-flotillin-1 (dilution 1:10,000, ab133497, Abcam). Subsequently, a specific HRP-conjugated secondary antibody (dilution 1:2000, Cell Signaling Technology, Danvers, MA, United States) was applied for detection. Positivity was visualized by applying substrates for the chemiluminescence reaction of HRP (Amersham ECL Prime Western Blotting Detection Reagent, GE Healthcare, Chicago, IL, United States) and capturing the reaction on photographic sheets through autoradiography (GE Healthcare).

Images were scanned using an Epson Perfection 1,260 scanner. Gel running was conducted under non-denatured conditions for the detection of CD81, and CD63, while denatured conditions were employed for other markers.

#### 2.7.3 Electron microscopy

For TEM analysis, EV preparations from MSCs and iMSCs were fixed by adding 2% paraformaldehyde (PFA) in 0.1 mol/L phosphate buffer (pH 7.4) in the same volume as the EV resuspension buffer. Five μl drops of EVs were then adsorbed for 10 min on formvar-carbon-coated copper grids. Subsequently, the grids were rinsed in PBS and negatively stained with 2% uranyl acetate for 5 min at room temperature. The stained grids were finally embedded in 2.5% methylcellulose to enhance preservation and air-dried before examination (Gorgun et al., 2021). Electron micrographs were obtained using a Hitachi 7,800 120 kV electron microscope (Hitachi, Tokyo, Japan) operating at 100 kV, equipped with a Megaview G3 digital camera and Radius software (EMSIS).

#### 2.7.4 Multiplex EV surface marker analysis

Analysis of surface protein expression on EVs and iEVs was performed using the MACSplex Exosome kit human (Milteny Biotec), following the overnight protocol for detection of 37 different markers on the EVs surface (CD1c, CD2, CD3, CD4, CD8, CD9, CD11c, CD14, CD19, CD20, CD24, CD25, CD29, CD31, CD40, CD41b, CD42a, CD44, CD45, CD49e, CD56, CD62p, CD63, CD69, CD81, CD86, CD105, CD133.1, CD142, CD146, CD209, CD326, HLA-ABC, HLA-DR DP DQ, MCSP, ROR1 and SSEA-4), and two isotype controls (mIgG1 and REA control).

Briefly, 4 µg of EVs quantified by BCA assay were diluted in 120 µL of MACSPlex buffer and incubated with capture beads, coated with specific antibodies, overnight at room temperature with gentle agitation. Beads were then centrifuged and washed with MACSPlex buffer. Finally, a detection antibody mixture (CD9, CD63, CD81-APC conjugated antibody) was added and incubated for 1 h at room temperature with gentle agitation. Beads were centrifuged, washed with MACSplex buffer, and analyzed on a CytoFLEX Flow Cytometer (Beckman Coulter); collected data were then analyzed with FlowJo software (BD Bioscience). A PBS negative control was also carried out to analyze background with values obtained subtracted from the median of fluorescence (MFI) of the stained samples. APC fluorescence median values were then normalized to the mean signal of MFI for CD9, CD63, and CD81, resulting in normalized MFI values. For all the markers only proteins with a normalized MFI > 20 were considered for further analysis.

### 2.8 RNA extraction and real-time quantitative reverse transcription polymerase chain reaction (qRT-PCR)

Confluent hACs (P2) were treated for 16 h with 200 U/mL interleukin-1 alpha (IL-1α) ± 1 μg/mL of EVs or iEVs. Negative controls without IL-1α or EVs and positive controls treated only with 200 U/mL IL-1α were also included. Cell monolayers were harvested in TRIzol Reagent (Thermo Fisher Scientific, Waltham, MA, United States), and total RNA was extracted following the manufacturer’s instructions.

The concentration and purity of RNA were assessed using a BioSpectrometer (Eppendorf, Hamburg, Germany). With RNA quality confirmed by measuring the A260/A280 and A260/A230 ratios. Complementary DNA (cDNA) was synthesized from 2 μg of total RNA using SuperScript™ IV VILO™ Master Mix (Thermo Fisher Scientific, Waltham, MA, United States) according to the manufacturer’s instructions. Transcript levels of IL-6, IL-8, COX-2 genes were measured by real-time quantitative PCR (qRT-PCR) using BlasTaq™ 2X qPCR MasterMix (abmgood, Richmond, BC, Canada) on a 7,500 Fast Real-Time PCR System (Applied Biosystems, Foster City, CA, United States).

The housekeeping gene GAPDH was utilized as the endogenous control for normalization. The selected human-specific primer sequences are provided in Table 1. Data were analyzed using the 2^−ΔΔCT^ method and expressed as fold change relative to GAPDH. To ensure specificity and quality of the amplification, melt curve analysis was performed at the end of each qRT-PCR run. No-reverse transcriptase (NRT) controls and no-template controls (NTC) were included in all reactions to rule out contamination. Only samples with a single, specific melt peak were considered for further analysis.

**TABLE 1:** Primers used for quantitative real-time PCR.

	Forward primer	Reverse primer
*COX-2*	5′-AAT​TGC​TGG​CAG​GGT​TGC​T-3′	5′-GGT​CAA​TGG​AAG​CCT​GTG​ATA​CT-3′
*GAPDH*	5′-CCA​TCT​TCC​AGG​AGC​GAG​AT-3′	5′-CTG​CTT​CAC​CAC​CTT​CTT​GAT-3′
*IL-6*	5′-TGA​CGA​CCT​AAG​CTG​CAC​TT-3′	5′-GGG​CTG​ATT​GGA​AAC​CTT​ATT​A-3′
*IL-8*	5′-CGT​GGC​TCT​CTT​GGC​AGC-3′	5′-TTA​GCA​CTC​CTT​GGC​AAA​ACT​G-3′

### 2.9 Statistical analysis

Data were analyzed by One-way ANOVA using GraphPad Prism 9.3 software (GraphPad Software, Inc., San Diego, CA, United States). Levels of significance were set at p < 0.05 (*p < 0.05, **p < 0.01, ***p < 0.001, ****p < 0.0001). The number of data used for the statistical analyses is reported in the figure legends and corresponds to independent experiments. Data are shown as mean ± SD.

## 3 Results

### 3.1 MSC showed an increase in senescence rate during long-term *in vitro* expansion

A crucial limitation in therapeutic application of MSCs is represented by their low amount in all tissues and the quantity of isolated MSCs being insufficient for clinical use (Hassan et al., 2020). Large-scale *in vitro* expansion is required to meet the clinical demand. However, this can affect the cell quality as long-term expanded MSCs are reported to lose most of their stem cell features (Bonab et al., 2006).

On this basis, we aimed to determine the optimal range for MSC biological functionality, during long-term expansion in a xeno-free supplement (XFS). Human MSCs were cultured in the XFS-containing medium for 10 passages (P) (∼63 days), and characterized for their proliferation ability, senescence rate, and differentiation capacity.

**FIGURE 1:**
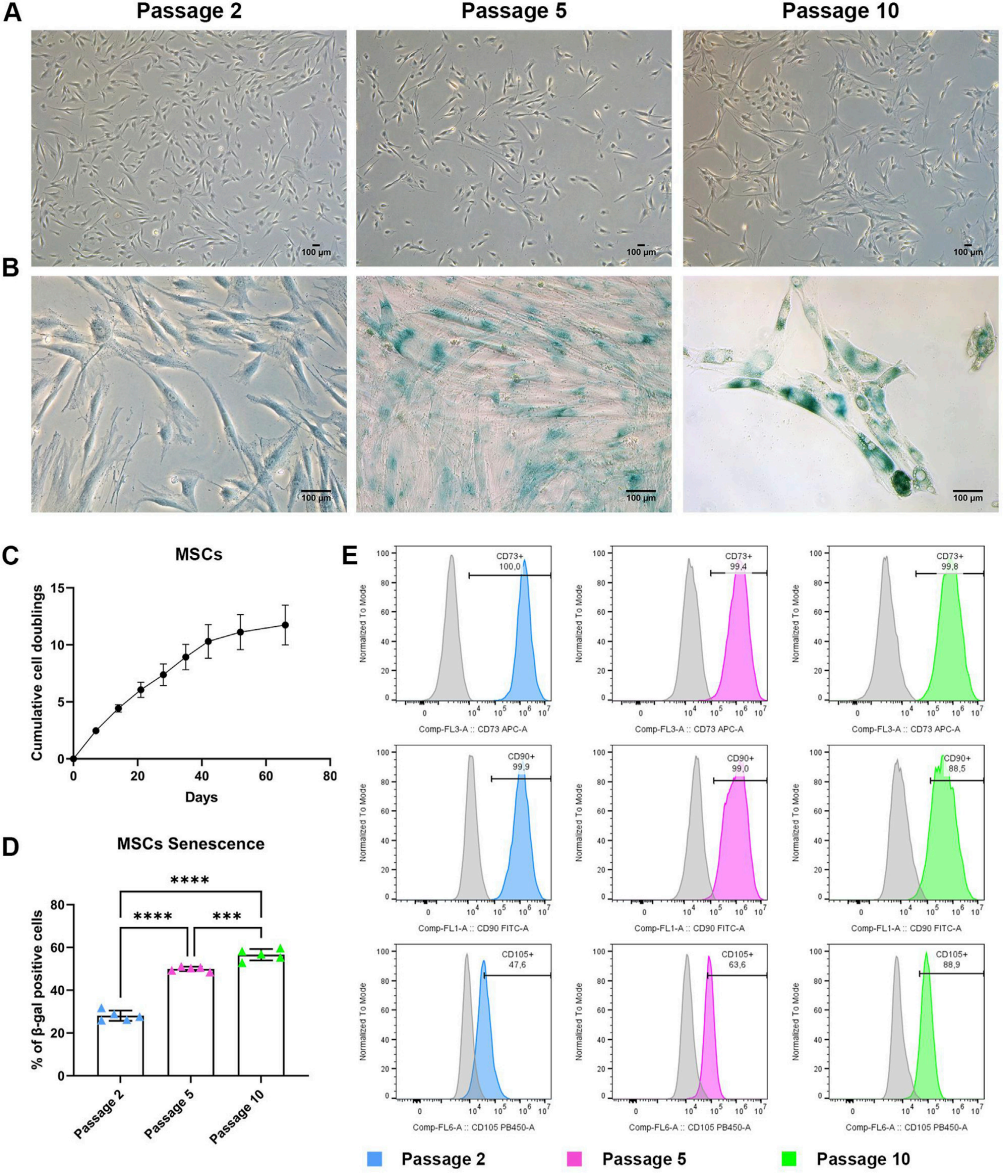
BMSC characterization during long term expansion **(A)** Representative images of MSCs at P2, P5, and P10. Scale bar = 100 μm. **(B)** Representative images of MSCs stained for senescence-associated β-galactosidase at P2, P5 and P10. Scale bar = 100 μm. **(C)** Growth curve of MSCs showing cumulative population doubling time. Data are represented as the mean ± SD, N = 3. **(D)** Quantification of senescence-associated β-galactosidase positive cells. Data are represented as the mean ± SD, N = 5, One-way ANOVA, *** *p-value* 0.001, **** *p-value* < 0.0001. **(E)** Representative histograms reporting flow cytometry analysis of MSCs at P2, P5 and P10. Histograms curve identify cells reacting CD73^−^, CD90^−^and CD105-specific antibodies. The area under the grey curve identifies cells reacting with the correspondent non-reactive immunoglobulin of the same type (isotype control). Data are representative of three independent experiments.

MSCs exhibited a typical fibroblast-like spindle-shaped morphology for the entire culture from P2 to P10 (Figure 1A). Despite this, high passage MSCs showed a more enlarged and flattened morphology compared to early culture passages.

Population doubling time allows assessment of MSC proliferation during *in vitro* culture. Human MSCs from three different donors were analysed for their growth kinetics by direct calculation of cumulative cell doublings from P2 to P10. Cells showed an exponential increase of cumulative cell doublings until P7 (∼35 days), reaching almost 8 doublings. At later passages the growth rate was reduced, and cell proliferation showed a progressive slowdown from P7 to P10 (Figure 1C). Cells reached 11.5 doublings at the end of the culture.

During long-term *in vitro* expansion, MSCs can proliferate for a limited number of doublings, before undergoing senescence and entering a growth-arrested stage. The impact of long-term expansion on senescence rate was evaluated for three different primary cultures by assessing MSCs β-galactosidase activity at P2 (dt 2.5), P5 (dt 7) and P10 (dt 11.5). The percentage of β-galactosidase positive MSCs increased from early to late passages (Figure 1B). MSCs at P2 showed a small percentage of β-galactosidase-positive cells, while P5 and P10 cultures exhibited more than 50% of senescent cells (Figure 1D).

Phenotypic characterization was also performed, as an essential step during MSC expansion for therapeutic purposes. MSCs expanded in XFS-containing medium were evaluated at P1, P5 and P10 (Figure 1E) with MSC surface expression of CD73, CD90 and CD105 (antigens typically associated to mesenchymal stromal cells) unaltered from early to late passages. In particular, the immunophenotypic analysis of P10 MSCs exhibited very high expression of CD73 and CD90 (>99,8% and 88,5%, respectively), even if cells were cultured for a long period of time (more than 60 days). CD31, CD34 and CD45 exclusion markers, which are normally not present on mesenchymal cells, were not expressed at P1, P5 nor P10.

These results suggested that long-term expansion of MSCs did not impact their phenotypic profile but was strongly associated with an increase in senescence over time.

### 3.2 MSCs showed a decrease in trilineage differentiation potential during long-term *in vitro* expansion

The ability of MSCs to differentiate toward cell types of the mesodermal lineages is progressively reduced when cells are expanded in culture (Banfi et al., 2000). MSCs osteogenic, adipogenic and chondrogenic differentiation capacity was investigated at P1 (dt 1.5), P5 (dt 7) and P10 (dt 11.5) for three different primary cultures.

**FIGURE 2:**
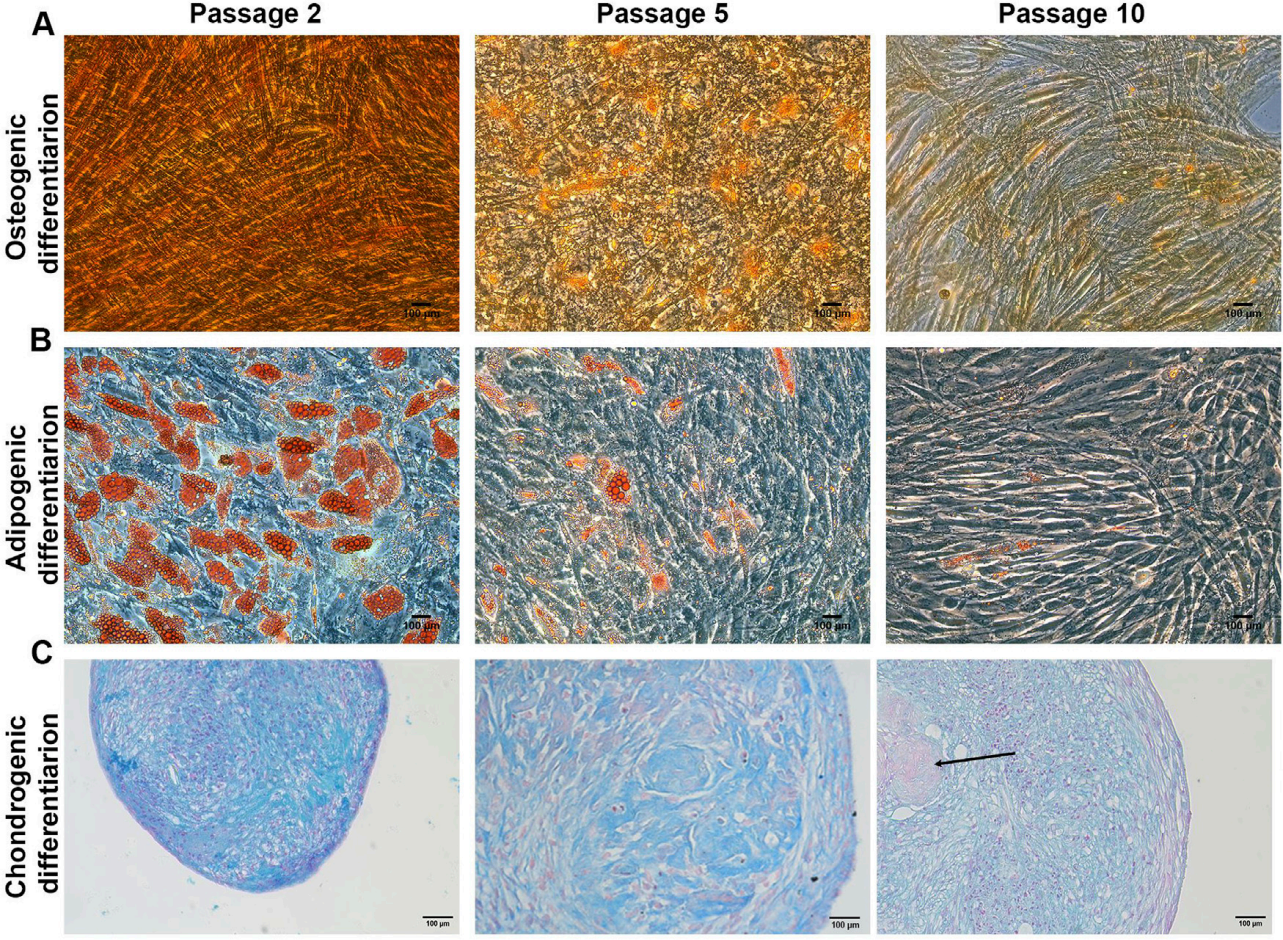
MSCs trilineage differentiation Representative images of **(A)** Alizarin red staining for osteogenic differentiation **(B)** Oil red O staining for adipogenic differentiation and **(C)** Alcian blue staining for chondrogenic differentiation. Scale bar = 100 μm (N = 3). Arrows indicate the fibrotic core.

During long-term expansion of MSCs in XFS-containing medium, the osteogenic potential showed an evident reduction from early to late passages (Figure 2A) with a gradual reduction of red calcium deposits observed from P1 to P10 cell preparations. These results reflect the lowering of MSCs osteogenic potential from early to late passages. MSC differentiation capacity towards adipocyte-like cells and consequent production of small lipid droplets showed a similar trend of reduction from early to late passages (Figure 2B). However, MSCs maintained their osteogenic and adipogenic differentiation potential for the entire expansion period, although reduced with time.

Chondrogenic potential of MSCs was also evaluated as the capacity of the cells to form a micro-mass pellet that differentiates to a cartilage-like tissue (Figure 2C). Interestingly, MSCs maintained their ability to form pellets for the entire culture through passages with Alcian Blue staining performed to evaluate the glycosaminoglycan deposition. While P2 MSCs-derived pellets showed a high percentage of positive staining, glycosaminoglycans deposition had decreased considerably in P5 MSCs-derived pellets and more so at P10. Furthermore, late stage P10 pellets also showed the presence of a fibrotic, Alcian blue negative core, as shown in Figure 2C.

### 3.3 XFS-containing medium provides long-term expansion of iMSCs compared to primary MSC cultures

Despite their potential, limitations in maintaining MSC cell consistency over long-term culture have limited the promise of MSC therapies (Mastrolia et al., 2019). The generation of induced pluripotent stem cell (iPSC)-derived MSC (iMSC) has the potential to overcome the limitations of adult MSCs for clinical applications (Lee et al., 2023). However, comprehensive research on the molecular and functional heterogeneity of iMSC is required.

**FIGURE 3:**
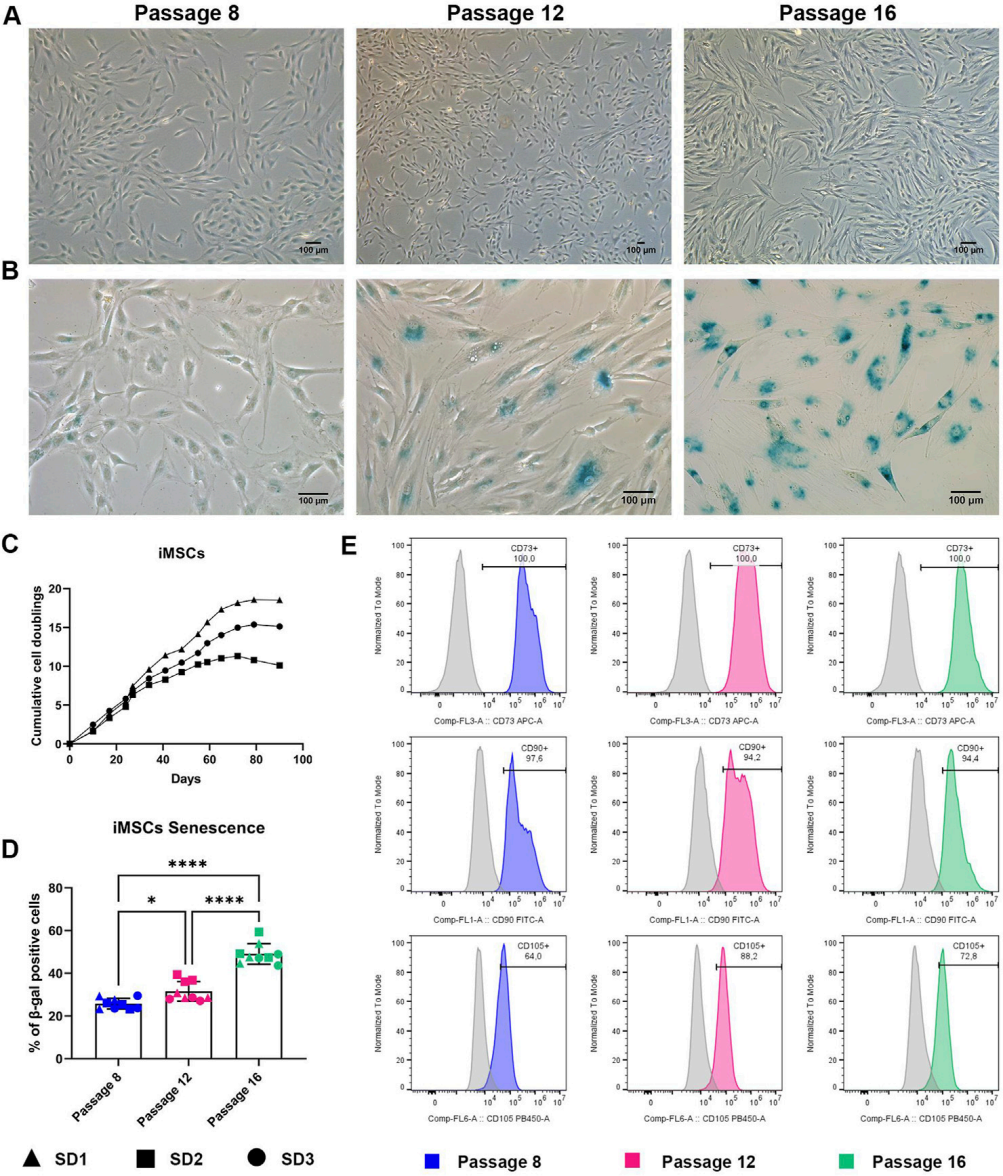
iMSC characterization during long term expansion **(A)** Representative images of iMSC at P8, P12 and P16. Scale bar = 100 μm. **(B)** Representative images of iMSC stained for senescence-associated β-galactosidase at P8, P12 and P16. Scale bar = 100 μm. **(C)** Growth curve of iMSCs showing cumulative population doubling time. Data are represented as the mean ± SD, N = 3. **(D)** Quantification of senescence-associated β-galactosidase positive cells. Data are represented as the mean ± SD, N = 5, One-way ANOVA, * *p-value* 0.05, **** *p-value* < 0.0001. **(E)** Representative histograms reporting flow cytometry analysis of iMSC at P8, P12 and P16. Histograms curve identify cells reacting CD73, CD90 and CD105-specific antibody. The area under the grey curve identifies cells reacting with the correspondent non-reactive immunoglobulin of the same type (isotype control). Data are representative of three independent experiments.

Three iMSC lines, derived from iPSC differentiation experiments (SD1, SD2 and SD3) were expanded in XFS-containing medium from P8 to P19 in a continuous long-term culture (∼100 days). Cell morphology of iMSCs displayed some changes during the culture. Although, iMSCs exhibited the classical spindle-shaped, fibroblastic-like morphology, in earlier passages, the cells had a less elongated, small, and poorly branched morphology at late passage where the iMSCs had increased in size (Figure 3A).

Although, the cumulative cell doubling rate in the first 35–40 days of cell expansion was very similar in all iMSC preparations (Figure 3C), a slight difference in the cell proliferation rate between iMSC batches was observed. Cell growth was continuous until 60 days (P15), allowing a consistent *in vitro* expansion of iMSCs for a longer time compared to primary MSCs cultures. However, after 60 days, cell growth reached a plateau.

Furthermore, iMSCs manifested high senescence-associated β-galactosidase activity at P16, correlating with the reduction of proliferation capability in comparison to early passages (Figure 3B). These results reflect the expected behaviour of aged-MSCs, suggesting that long-term expansion of these cells is associated with a decline of their stem properties. However, despite becoming senescent at higher passages, iMSCs outcompete MSCs in their capacity to be expanded in XFS-containing medium. In addition, a different behaviour between iMSCs batches was appreciated. SD2 iMSCs showed higher senescence levels, compared to both SD1 and SD3 iMSCs (Figure 3D).

Finally, the cell surface phenotype of iMSCs from early passages was compared with iMSCs at late passages. To this end, we investigated the expression of commonly used positive MSC markers following labelling with fluorochrome conjugated antibodies by flow cytometry. SD1, SD2 and SD3 at P8 (dt 6), P12 (dt 10.5) and P16 (dt 15) were assessed for the expression of CD73, CD90 and CD105 without showing any significant differences between early, middle, and late passaged iMSCs (Figure 3E). These results suggested that iMSCs can be successfully expanded in XFS-containing medium without changing MSC characteristics.

### 3.4 iMSC lines showed a heterogeneous differentiation potential during long-term *in vitro* expansion

**FIGURE 4:**
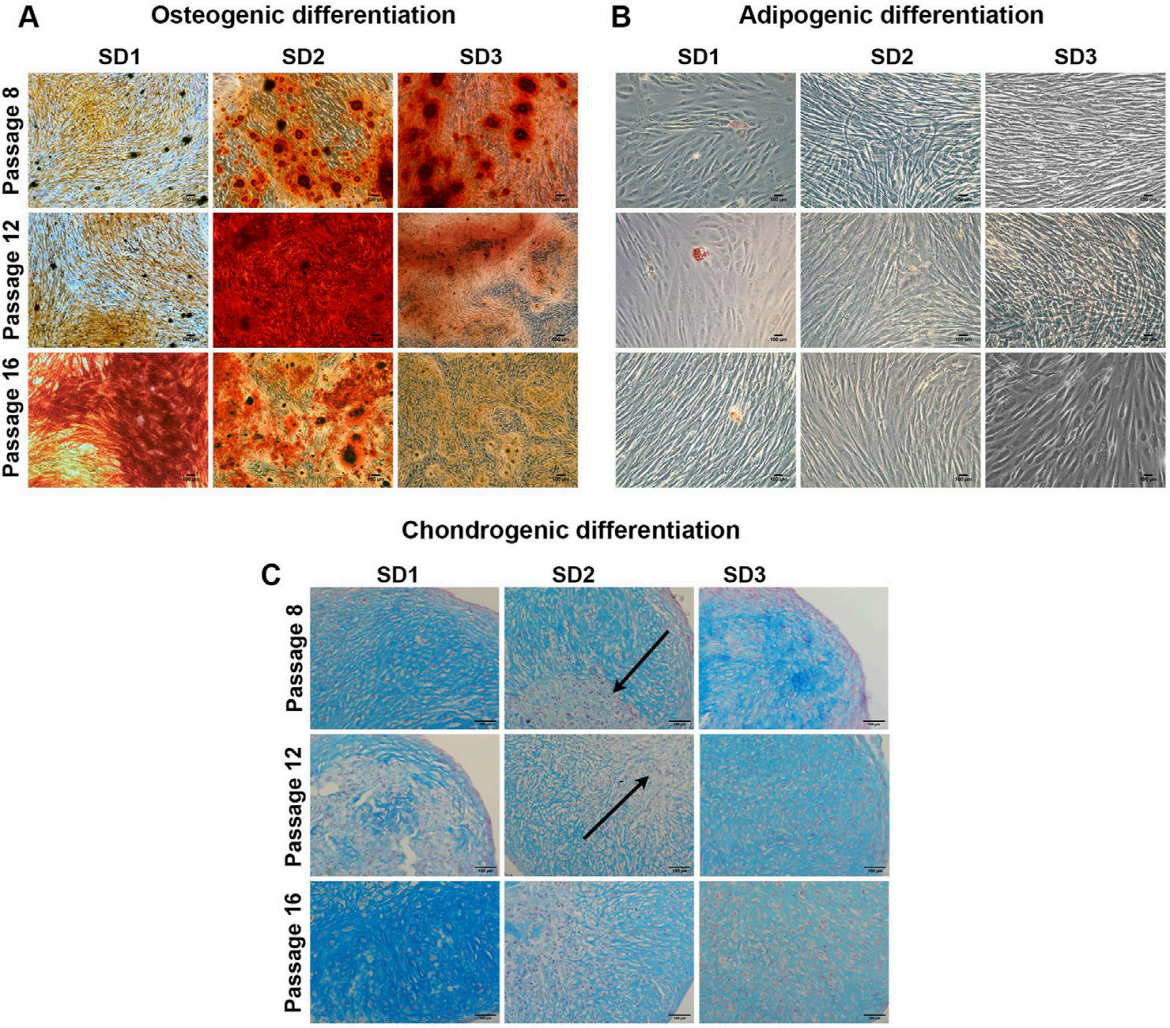
iMSC trilineage differentiation Representative images of **(A)** Alizarin red staining for osteogenic differentiation **(B)** Oil red O staining for adipogenic differentiation and **(C)** Alcian blue staining for chondrogenic differentiation. Scale bar = 100 μm (N = 3). Arrows indicate the fibrotic core.

Following expansion of iMSCs, their capability to differentiate *in vitro* along the osteogenic, adipogenic and chondrogenic lineages was investigated at P8 (dt 6), P12 (dt 10.5) and P16 (dt 15). Figure 4A shows osteogenic differentiation of iMSC lines, underlining a wide heterogeneity of iMSC differentiation capability between different lines and culture passages. SD1 showed very poor osteogenic differentiation at early (P8) and middle (P12) passages, while a strong calcium deposition was observed at late (P16) passage. SD2 also exhibited low osteogenic differentiation at early passages (P8), with a strong increase by P12, followed by a decrease in calcium deposition at late passage (P16). However, SD2 appeared to be a more bone-prone iMSC line compared to SD1 iMSCs while SD3 iMSCs showed strong osteogenic potential at early passages, but this decreased gradually during the culture period. Contrary, to MSC primary cultures, all the iMSC lines had low or no ability to differentiate to the adipogenic lineage (Figure 4B).

Chondrogenic potential showed a similar trend to the osteogenesis with the iMSCs, showing a wide heterogenicity. Alcian Blue staining was used to highlight glycosaminoglycan deposition in the ECM of the differentiating iMSC (Figure 4C). Similarly to osteogenic differentiation results, SD1 iMSCs exhibited a higher differentiation potential at late culture passages (P16), while SD3 showed high glycosaminoglycans deposition only at early (P8) passage. However, SD2 tended to form pellets with a fibrotic core and low Alcian Blue staining. These results suggested that different batches of iPSC derived MSCs may show widely different potential and every batch need to be investigated for quality before clinical use.

### 3.5 iMSCs secreted higher amounts of EVs compared to MSCs

Following cell characterisation, EVs were separated from the culture media of MSCs and iMSCs by differential ultracentrifugation at early, middle, and late passages. MSC-EVs (EVs) and iMSC-EVs (iEVs) were quantified by Nanoparticle Tracking Analysis (NTA).

**FIGURE 5:**
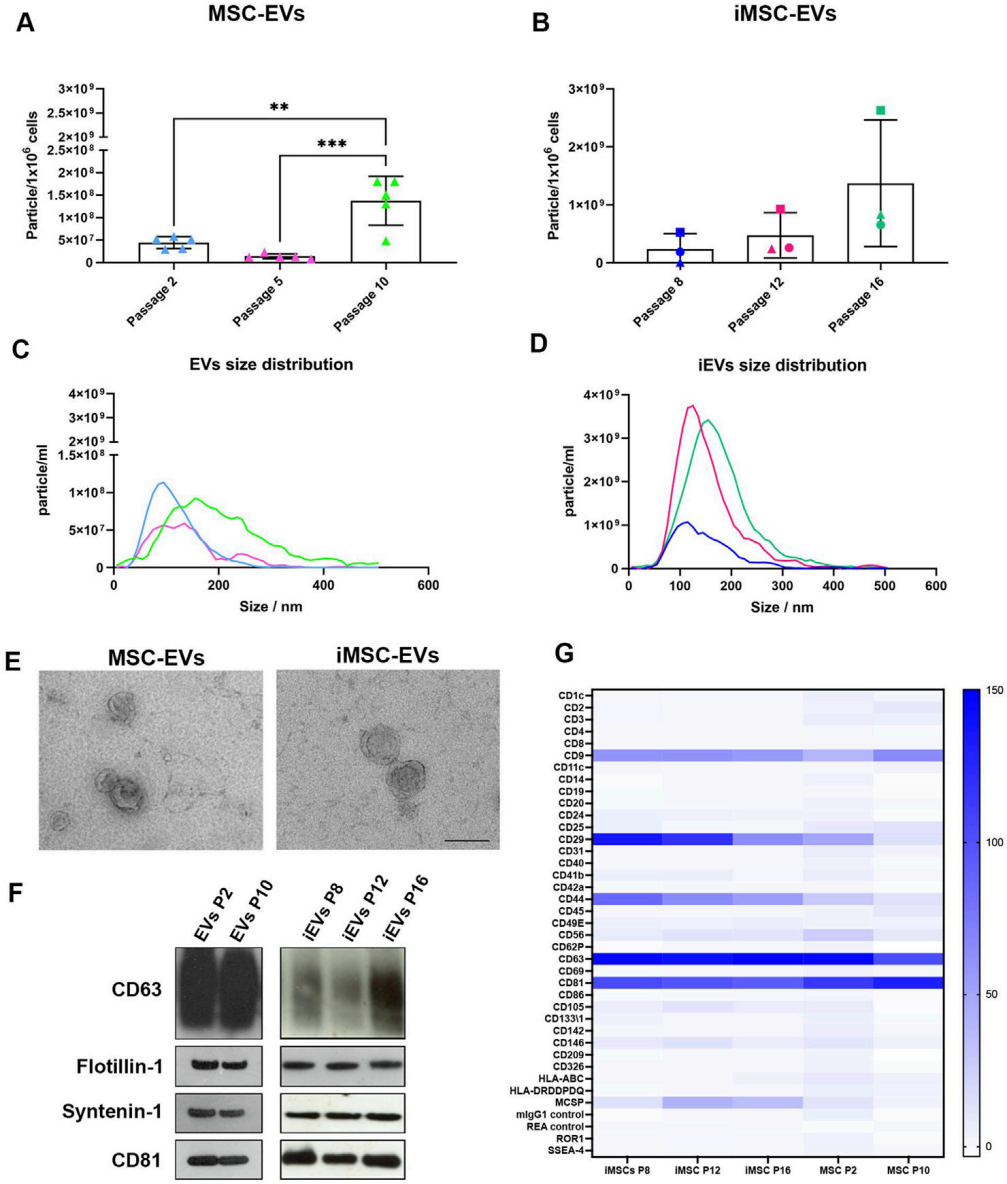
MSC and iMSC derived EVs characterization NTA of particle concentration of EVs **(A)** and iEVs **(B)**, expressed as particle/10^6^ cells. Data are represented as the mean ± SD; ** p-value <0.01, *** p-value <0.001 (N = 5), one-way ANOVA). Representative NTA size distribution analysis of EVs **(C)** and iEVs **(D)**. **(E)** TEM micrographs of isolated EVs and iEVs. Scale bar = 200 nm. **(F)** Western blot analysis on EVs and iEVs. Specific expression of CD63, CD81, flotillin-1 and syntenin-1. **(G)** Heatmap showing EVs and iEVs surface phenotype characterized using multiplex beads-based flow cytometry assay. Data are represented as CD9/CD63/CD81 normalized MFI, only protein with MFI > 20 were considered as expressed from the EVs. Colour scale identify CD9/CD63/CD81 normalized MFI, N = 3.

Quantification of both EV and iEVs preparations by NTA showed a significant increase in particle concentration through passages during long-term *in vitro* culture (Figures 5A, B). Significantly higher EV levels were observed in P10 EVs compared to both P2 and P5 EVs. Similarly to previous cell culture analyses, iMSCs batches showed high variability in levels of iEV secretion. However, all preparations exhibited a similar trend, with an increase in iEVs production at late (P16) passage compared to both middle (P12) and early (P8) passages.

Size distribution of both EVs and iEVs was also evaluated. Interestingly, heterogenicity of vesicle dimensions seemed to increase over time correlating with both MSCs and iMSC ageing (Figures 5C, D).

In addition, EVs and iEVs were also investigated by TEM to confirm their efficient isolation. Both EVs and iEVs exhibited the typical morphology of a vesicle surrounded by a lipidic bilayer, with a low level of clustering (Figure 5E). Western blot analysis of MSC and iMSC derived EVs (Figure 5F; Supplementary Figure S1) highlighted that both EVs and iEVs showed a similar phenotype concerning the expression of syntenin-1, flotillin-1, CD63 and CD81 among culture passages, suggesting that EVs maintained a consistent phenotype.

Further analysis of the EVs surface phenotype was performed by a multiplex beads-based flow cytometry assay (Figure 5G). The MFI of single markers was normalized to the mean of the three tetraspanin CD9, CD63 and CD81 markers with a normalized MFI > 20 defining expression on the EV surface. CD29 and CD44 were noted to be strongly expressed in iEVs, with a gradual decrease during culture passages. On the other hand, EVs exhibited a lower expression of both CD29 and CD44 markers at early and late passage. Moreover, a mid-expression of MCSP was also highlighted in iEVs at middle (P12) and late (P16) passages.

### 3.6 EVs and iEVs showed different anti-inflammatory effects on an *in vitro* OA model

**FIGURE 6:**
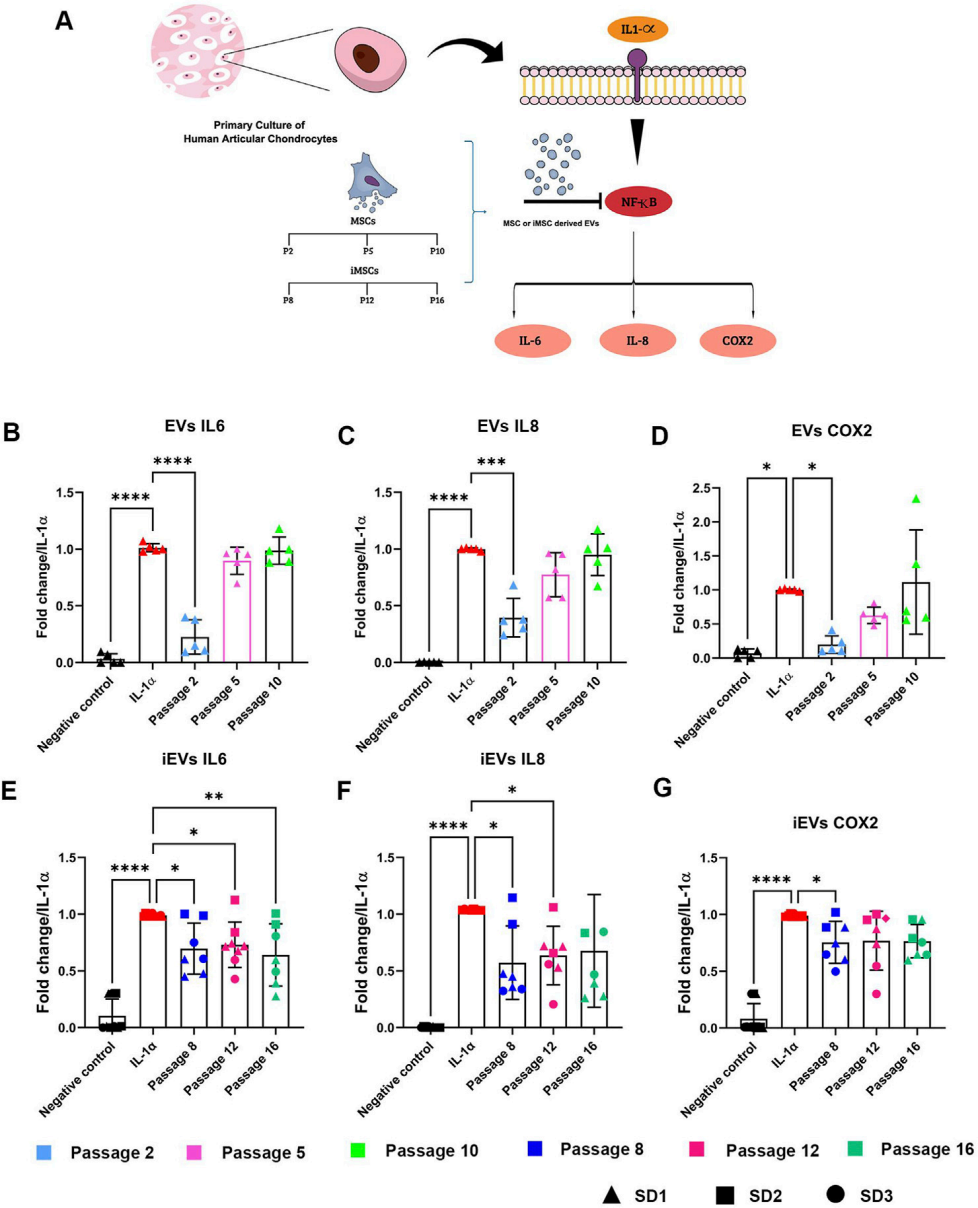
EVs and iEVs anti-inflammatory effects in an *in vitro* OA model **(A)** Scheme showing the effects of IL-1α treatment on hACs used to create an *in vitro* OA model. EV treatment is able to block the production of pro-inflammatory protein and reduce level of inflammation caused by IL-1α treatment. Expression levels of *IL-6, IL-8, COX-2* quantified with real-time PCR of hACs treated with IL-1α and EVs **(B–D)** or iEVs **(E–G)** derived from different culture passages. Data are represented as the mean ± SD; * *p-value* < 0,05, ** p-value <0.01, *** p-value <0.001, **** *p-value* < 0.0001 (N = 5, one-way ANOVA).

As the role of MSCs in attenuating local inflammation has generated high interest in the scientific community, the paracrine effect of MSCs and iMSCs was evaluated on osteoarthritic chondrocytes in an *in vitro* model (Figure 6A). Human articular chondrocytes were treated with IL-1α, to mimic the inflammatory process occurring in OA. Inflamed chondrocyte-mediated release of specific cytokines (IL-6, IL-8 and COX-2) was quantified after the administration of either EVs or iEVs derived from MSCs and iMSC respectively, at selected culture passages.

Anti-inflammatory effects of MSC-EVs were extremely dependent on the culture passage. In particular, EVs derived from early passaged MSCs exerted a strong anti-inflammatory effect, able to decrease the expression of the inflammatory mediators Il-6 (Figure 6B), IL-8 (Figure 6C) and COX-2 (Figure 6D). This anti-inflammatory effect drastically decreased with MSC ageing. For middle (P5) and late (P10) passages EVs, no significant changes in comparison to the positive control were observed.

On the other hand, iMSC-derived EVs showed an effect which could be considered as independent of the culture passage (Figures 6E–G). Mirroring the heterogeneity of parental cells, iEVs also showed high variability in anti-inflammatory properties among different batches. SD1-and SD3-derived iEVs were indeed able to exert a strong inhibitory effect on IL-6, IL-8 and COX-2 expression, while this effect was not achieved after administration of SD2-derived iEVs.

## 4 Discussion

MSCs and their EVs are widely recognized for regenerative medicine applications, especially in orthopaedics (Wang et al., 2023; Yin et al., 2022). Despite their properties, obtaining MSCs from donors presents challenges due to variations in behaviour depending on the donor, limited expansion potential, and difficulty in obtaining sufficient EV quantities for large-scale experiments (Maumus et al., 2020; Wiest and Zubair, 2020). As a result, researchers have begun to explore alternative sources for MSCs and their EVs (Cai et al., 2020). In previous studies, our research group demonstrated that MSC-EVs reflect the characteristics of their originating cells, such as angiogenic properties in adipose-derived MSCs (ADMSCs) and chondrogenic effects in bone marrow-derived MSCs (BMSCs) (Gorgun et al., 2021). Consequently, MSCs derived from human iPSCs appear as a promising cell source for the manufacturing of MSC-EV therapeutics (Bertolino et al., 2022). Hence, we hypothesized that EVs derived from iMSCs could address these challenges and serve as a superior source for bone marrow derived-MSC-EVs for the treatment of osteoarthritis.

To investigate this hypothesis, dynamic changes in MSCs during long-term *in vitro* expansion in XFS- containing medium over an extended passage period (∼63 days) were studied with a focus on their proliferation, senescence rate, and phenotypic profile at different passages. The morphological changes observed in MSCs during long-term culture, characterised by the enlargement of cells were consistent with the previous studies that showed that alterations in cellular morphology were associated with cellular senescence (Iwata et al., 2021; Yoon et al., 2014). Indeed, an exponential increase in MSC proliferation up to a certain passage was observed, followed by a decrease in the proliferative capacity. This correlated with elevated β-galactosidase activity detected over successive passages, indicating increased senescence rates at late passages. These changes likely reflect an underlying cellular aging processes that may impact the functional properties of MSCs, potentially influencing their therapeutic efficacy (Miclau et al., 2023). This reduction in proliferative capacity has important implications for the scalability of MSC production for clinical applications, as it may limit the yield of functional cells that can be obtained from a given starting population. Interestingly, as observed by Yan et al. who demonstrated that at least 96.6% of the MSC populations expressed CD29, CD44, CD73, CD90, and CD105 antigens regardless of culture conditions and passage number, we similarly observed the maintenance of MSC surface marker expression throughout long-term expansion, suggesting that the phenotypic profile of MSCs remained relatively stable despite the onset of senescence (Yang et al., 2018). However, it is important to note that changes in functional properties, such as differentiation potential and paracrine activity, may occur independently of alterations in surface marker expression, highlighting the need for comprehensive characterization of late passage MSC populations. Interestingly, despite the observed reductions in osteogenic and adipogenic potential, MSCs retained their ability to form chondrogenic micro-mass pellets throughout the culture passages. Nevertheless, closer examination revealed a decline in glycosaminoglycan deposition and the presence of a fibrotic core in late-stage pellets, indicating compromised chondrogenic capacity which limits the ability of late passage-MSCs to be used for osteoarthritis (Palamà et al., 2023).

Through comprehensive research involving three iMSC lines derived from iPSC differentiation experiments, we observed dynamic changes in cell morphology, proliferation kinetics, and senescence patterns during long-term culture. Despite exhibiting variations in growth kinetics and senescence levels among batches, iMSCs consistently demonstrated prolonged *in vitro* expansion compared to primary MSC cultures, as has been shown in previous studies (Lee et al., 2023; Spitzhorn et al., 2019). On the other hand, iMSCs maintained consistent expression of commonly used MSC markers like their counterparts, indicating their ability to preserve key mesenchymal characteristics throughout prolonged expansion. Generated iMSC can also be expanded in culture over an extended period without losing their typical fibroblastic morphology and their capacity for osteo-chondro progenitor differentiation. Interestingly, chondrogenic differentiation of iMSCs exhibited considerable heterogeneity among batches, with varying levels of glycosaminoglycan deposition in the extracellular matrix. These results emphasize the importance of thoroughly investigating the differentiation potential of each batch of iMSCs before considering their use in clinical applications.

Following cell characterization, we generated MSC and iMSC derived EV preparations to elucidate and compare their characteristics over time in culture. Our findings reveal a significant increase in EV secretion levels with the progression of culture passages, indicative of an accumulation of EVs over long-term culture in both cell types. However, senescence at late passages was also seen. Notably, iMSCs exhibited a similar trend of increasing EV production to MSCs, albeit with considerable variability among batches. Additionally, there was an enrichment of larger vesicles in late passages of both MSCs and iMSCs, potentially reflecting cellular aging processes. This finding underscores the importance of understanding the dynamic changes in EV properties over culture passages for optimizing their therapeutic efficacy (Tertel et al., 2023). Importantly, our characterization of EV morphology via transmission electron microscopy confirms the efficient isolation of EVs from both MSCs and iMSCs, validating their structural integrity. Additionally, western blot analysis and multiplex beads-based flow cytometry assays demonstrate the consistent expression of EV markers across culture passages, indicative of a stable EV phenotype.

Of particular interest is the differential expression of surface markers, such as CD29, CD44, and MCSP, which are highly expressed in iEVs compared to MSC-EVs. CD29, also known as Integrin Subunit Beta 1 (ITGB1), is a glycoprotein that is widely expressed by a variety of cells, including MSCs, and has been found in several types of EVs across a broad range of studies (Weber et al., 2023; Ekström et al., 2022). Similarly, CD44 is a transmembrane glycoprotein acting as a cell surface adhesion receptor, widely distributed in normal adult tissue but highly expressed in several types of EVs from different tissues (Ramos T et al., 2016). Even though CD29 and CD44 expression is high in MSCs derived EVs, their differential expression in iEVs highlights the unique properties of iMSCs (Pavathuparambil Abdul Manaph et al., 2019). Interestingly, Melanoma-Associated Chondroitin Sulfate Proteoglycan (MCSP) is also found to be expressed by chondrocytes in articular cartilage, where play an important role in mechanical cartilage homeostasis and in the initiation and progression of OA (Krug et al., 2015).

This expression pattern could present an advantage of using XFS media for the culture of iMSCs to obtain iEVs for the treatment of osteoarthritis.

Since the previous studies have already demonstrated that hBMSCs and hiMSCs release EVs with similar immunomodulatory properties *in vitro*, tested in a multi-donor mixed lymphocyte reaction (mdMLR) assay (Ramos et al., 2022), we wanted to further analyse the biological activity of the EVs and iEVs on osteoarthritic chondrocytes using an IL-1α-induced *in vitro* osteoarthritis model (Palamà et al., 2020). Our results demonstrated that MSC-EVs exerted a strong anti-inflammatory effect, particularly when derived from early-passaged MSCs. However, this anti-inflammatory effect diminished with MSC aging, suggesting a passage-dependent modulation of EV-mediated immunomodulation. Despite the lack of adipogenic differentiation capability, obtained hiMSC-EV samples show biological activity in suppressing secretion of pro-inflammatory IL-8 by human articular chondrocytes even if prepared from conditioned media of late passaged cells. Moreover, the anti-inflammatory effect of iMSC-derived EVs appears to be independent of culture passage, highlighting their potential as a consistent therapeutic option for osteoarthritis. Nonetheless, our findings also reveal significant variability in the anti-inflammatory properties of iMSC-derived EVs among different batches, reflecting the heterogeneity of parental iMSCs. This underlines the importance of further investigations to optimize the therapeutic efficacy and consistency of iMSC-derived EVs for osteoarthritis treatment.

## 5 Conclusion

Taken together, results suggested that the effects of iMSC-EVs are prolonged compared to MSC-EVs which rapidly lose their effects after the first culture passages, providing a larger window of activity. However, the variability and heterogeneity of iMSC differentiation batches represent a great limitation in the use of iMSC-derived EVs as a potential tool in clinical treatment of OA. This variability in iMSC differentiation batches could impact the consistency and efficacy of the EVs produced from them, making it challenging to standardize their use for clinical treatment of OA. It is crucial to address these limitations through further research and potentially develop methods to minimize batch-to-batch variability and enhance the reliability and effectiveness of iMSC-derived EVs for OA treatment.

## Data Availability

The raw data supporting the conclusions of this article will be made available by the authors, without undue reservation.

## Supplementary material

SUPPLEMENTARY FIGURE 1S Uncropped Western Blots images of Figure 5F: Unmodified western blot images from Figure 5F with molecular weight standard **(A)** Detection of specific vesicular markers on MSC-EVs at different time points (passage 2 and 10). **(B)** Detection of specific vesicular markers on iMSC-EVs at different time points (passage 8, 12, and 16); in order for each lane are: Ladder, CD81 (25 kDA), CD 63 (63 kDa), Flotillin (48 kDA) and Syntenin (35 kDA). The red frames indicate the part cut to reproduce Figure 5F.

## Abbreviations

iPSC, induced Pluripotent Stem Cells (iPSC); MSCs, Mesenchymal Stromal Cells; MSC-EVs, Mesenchymal Stromal Cell-derived Extracellular Vesicles; iMSCs, induced Pluripotent Stem Cells-derived Mesenchymal Stromal Cells; OA, Osteoarthritis; XFS, Xeno-Free; EVs, Extracellular Vesicles; iEVs, induced Pluripotent Stem Cells-derived Mesenchymal Stromal Cells- Extracellular Vesicles; P, Passage number; PFA, Paraformaldehyde; CM, Conditioned Medium; PBS, Phosphate-Buffered Saline; ADMSCs, Adipose-derived Mesenchymal Stromal Cells; BMSCs, Bone marrow-derived Mesenchymal Stromal Cells.

## References

[B1] Abdal D. A. Lee S. B. Kim K. Lim K. M. Jeon T. i. Seok J. (2019). Production of mesenchymal stem cells through stem cell reprogramming. Int. J. Mol. Sci. 20, 1922. 10.3390/ijms20081922 31003536 PMC6514654

[B2] Banfi A. Muraglia A. Dozin B. Mastrogiacomo M. Cancedda R. Quarto R. (2000). Proliferation kinetics and differentiation potential of *ex vivo* expanded human bone marrow stromal cells: implications for their use in cell therapy. Exp. Hematol. 28, 707–715. 10.1016/s0301-472x(00)00160-0 10880757

[B3] Bertolino G. M. Maumus M. Jorgensen C. Noël D. (2022). Recent advances in extracellular vesicle-based therapies using induced pluripotent stem cell-derived mesenchymal stromal cells. Biomedicines 10, 2281. 10.3390/biomedicines10092281 36140386 PMC9496279

[B4] Bjørge I. Kim S. Mano J. Kalionis B. Chrzanowski W. (2018). Extracellular vesicles, exosomes and shedding vesicles in regenerative medicine–a new paradigm for tissue repair. Biomaterials Sci. 6, 60–78. 10.1039/c7bm00479f 29184934

[B5] Bonab M. M. Alimoghaddam K. Talebian F. Ghaffari S. H. Ghavamzadeh A. Nikbin B. (2006). Aging of mesenchymal stem cell *in vitro* . BMC cell Biol. 7, 14–17. 10.1186/1471-2121-7-14 16529651 PMC1435883

[B6] Cai J. Wu J. Wang J. Li Y. Hu X. Luo S. (2020). Extracellular vesicles derived from different sources of mesenchymal stem cells: therapeutic effects and translational potential. Cell and Biosci. 10, 69–14. 10.1186/s13578-020-00427-x PMC7245623 32483483

[B7] Coryell P. R. Diekman B. O. Loeser R. F. (2021). Mechanisms and therapeutic implications of cellular senescence in osteoarthritis. Nat. Rev. Rheumatol. 17, 47–57. 10.1038/s41584-020-00533-7 33208917 PMC8035495

[B8] Diederichs S. Tuan R. S. (2014). Functional comparison of human-induced pluripotent stem cell-derived mesenchymal cells and bone marrow-derived mesenchymal stromal cells from the same donor. Stem cells Dev. 23, 1594–1610. 10.1089/scd.2013.0477 24625206 PMC4086513

[B9] Ekström K. Crescitelli R. Pétursson H. I. Johansson J. Lässer C. Olofsson Bagge R. (2022). Characterization of surface markers on extracellular vesicles isolated from lymphatic exudate from patients with breast cancer. BMC cancer 22, 50–17. 10.1186/s12885-021-08870-w 35012489 PMC8744234

[B10] Gorgun C. Africano C. Ciferri M. C. Bertola N. Reverberi D. Quarto R. (2022). Preconditioned mesenchymal stromal cell-derived extracellular vesicles (EVs) counteract inflammaging. Cells 11, 3695. 10.3390/cells11223695 36429124 PMC9688039

[B11] Gorgun C. Palamà M. E. F. Reverberi D. Gagliani M. C. Cortese K. Tasso R. (2021). Role of extracellular vesicles from adipose tissue-and bone marrow-mesenchymal stromal cells in endothelial proliferation and chondrogenesis. Stem Cells Transl. Med. 10, 1680–1695. 10.1002/sctm.21-0107 34480533 PMC8641083

[B12] Hassan MNFB Yazid M. D. Yunus M. H. M. Chowdhury S. R. Lokanathan Y. Idrus R. B. H. (2020). Large-scale expansion of human mesenchymal stem cells. Stem Cells Int. 2020, 1–17. 10.1155/2020/9529465 PMC7378617 32733574

[B13] Hong S. Kim H. Kim J. Park T. S. Kim T. M. (2024). Extracellular vesicles from induced pluripotent stem cell-derived mesenchymal stem cells enhance the recovery of acute kidney injury. Cytotherapy 26, 51–62. 10.1016/j.jcyt.2023.09.003 37843481

[B14] Iwata T. Mizuno N. Ishida S. Kajiya M. Nagahara T. Kaneda-Ikeda E. (2021). Functional regulatory mechanisms underlying bone marrow mesenchymal stem cell senescence during cell passages. Cell Biochem. Biophysics 79, 321–336. 10.1007/s12013-021-00969-y 33559812

[B15] Kraus V. B. Blanco F. J. Englund M. Karsdal M. Lohmander L. (2015). Call for standardized definitions of osteoarthritis and risk stratification for clinical trials and clinical use. Osteoarthr. Cartil. 23, 1233–1241. 10.1016/j.joca.2015.03.036 PMC4516635 25865392

[B16] Krug C. Birkholz K. Paulus A. Schwenkert M. Schmidt P. Hoffmann N. (2015). Stability and activity of MCSP-specific chimeric antigen receptors (CARs) depend on the scFv antigen-binding domain and the protein backbone. Cancer Immunol. Immunother. 64, 1623–1635. 10.1007/s00262-015-1767-4 26515978 PMC11028909

[B17] Lee H.-R. Kim S. Shin S. Jeong S. Y. Lee D. W. Lim S. U. (2023). iPSC-derived MSCs are a distinct entity of MSCs with higher therapeutic potential than their donor-matched parental MSCs. Int. J. Mol. Sci. 24, 881. 10.3390/ijms24010881 36614321 PMC9821152

[B18] Levy D. Abadchi S. N. Shababi N. Ravari M. R. Pirolli N. H. Bergeron C. (2023). Induced pluripotent stem cell‐derived extracellular vesicles promote wound repair in a diabetic mouse model via an anti‐inflammatory immunomodulatory mechanism. Adv. Healthc. Mater. 12, 2300879. 10.1002/adhm.202300879 37335811 PMC10592465

[B19] Li J. J. Hosseini-Beheshti E. Grau G. E. Zreiqat H. Little C. B. (2019). Stem cell-derived extracellular vesicles for treating joint injury and osteoarthritis. Nanomaterials 9, 261. 10.3390/nano9020261 30769853 PMC6409698

[B20] Martin I. Galipeau J. Kessler C. Le Blanc K. Dazzi F. (2019). Challenges for mesenchymal stromal cell therapies. Sci. Transl. Med. 11, eaat2189. 10.1126/scitranslmed.aat2189 30787168

[B21] Mastrolia I. Foppiani E. M. Murgia A. Candini O. Samarelli A. V. Grisendi G. (2019). Challenges in clinical development of mesenchymal stromal/stem cells: concise review. Stem cells Transl. Med. 8, 1135–1148. 10.1002/sctm.19-0044 31313507 PMC6811694

[B22] Maumus M. Rozier P. Boulestreau J. Jorgensen C. Noël D. (2020). Mesenchymal stem cell-derived extracellular vesicles: opportunities and challenges for clinical translation. Front. Bioeng. Biotechnol. 8, 997. 10.3389/fbioe.2020.00997 33015001 PMC7511661

[B23] Meng W.-T. Guo H.-D. (2023). Small extracellular vesicles derived from induced pluripotent stem cells in the treatment of myocardial injury. Int. J. Mol. Sci. 24, 4577. 10.3390/ijms24054577 36902008 PMC10003569

[B24] Miclau K. Hambright W. S. Huard J. Stoddart M. J. Bahney C. S. (2023). Cellular expansion of MSCs: shifting the regenerative potential. Aging Cell 22, e13759. 10.1111/acel.13759 36536521 PMC9835588

[B25] Palamà M. E. F. Coco S. Shaw G. M. Reverberi D. Ghelardoni M. Ostano P. (2023). Xeno-free cultured mesenchymal stromal cells release extracellular vesicles with a “therapeutic” miRNA cargo ameliorating cartilage inflammation *in vitro* . Theranostics 13, 1470–1489. 10.7150/thno.77597 37056573 PMC10086204

[B26] Palamà M. E. F. Shaw G. M. Carluccio S. Reverberi D. Sercia L. Persano L. (2020). The secretome derived from mesenchymal stromal cells cultured in a xeno-free medium promotes human cartilage recovery *in vitro* . Front. Bioeng. Biotechnol. 8, 90. 10.3389/fbioe.2020.00090 32117953 PMC7033421

[B27] Pavathuparambil Abdul Manaph N. Sivanathan K. N. Nitschke J. Zhou X. F. Coates P. T. Drogemuller C. J. (2019). An overview on small molecule-induced differentiation of mesenchymal stem cells into beta cells for diabetic therapy. Stem cell Res. and Ther. 10, 293–311. 10.1186/s13287-019-1396-5 31547868 PMC6757413

[B28] Ramos Y. F. Tertel T. Shaw G. Staubach S. de Almeida R. C. Suchiman E. (2022). Characterizing the secretome of licensed hiPSC-derived MSCs. Stem Cell Res. and Ther. 13, 434. 10.1186/s13287-022-03117-2 36056373 PMC9438242

[B29] Ramos T L. Sánchez-Abarca L. I. Muntión S. Preciado S. Puig N. Lopez-Ruano G. (2016). MSC surface markers (CD44, CD73, and CD90) can identify human MSC-derived extracellular vesicles by conventional flow cytometry. Cell Commun. Signal. 14, 1–14. 10.1186/s12964-015-0124-8 26754424 PMC4709865

[B30] Sabapathy V. Kumar S. (2016). Hi PSC‐derived iMSC s: NextGen MSC s as an advanced therapeutically active cell resource for regenerative medicine. J. Cell. Mol. Med. 20, 1571–1588. 10.1111/jcmm.12839 27097531 PMC4956943

[B31] Spitzhorn L.-S. Megges M. Wruck W. Rahman M. S. Otte J. Degistirici Ö. (2019). Human iPSC-derived MSCs (iMSCs) from aged individuals acquire a rejuvenation signature. Stem cell Res. and Ther. 10, 100–118. 10.1186/s13287-019-1209-x 30885246 PMC6423778

[B32] Stenderup K. Justesen J. Clausen C. (2003). Aging is associated with decreased maximal life span and accelerated senescence of bone marrow stromal cells. Bone 33, 919–926. 10.1016/j.bone.2003.07.005 14678851

[B33] Tertel T. Dittrich R. Arsène P. Jensen A. Giebel B. (2023). EV products obtained from iPSC-derived MSCs show batch-to-batch variations in their ability to modulate allogeneic immune responses *in vitro* . Front. Cell Dev. Biol. 11, 1282860. 10.3389/fcell.2023.1282860 37965578 PMC10642442

[B34] Wang X. Tian H. Yang X. Zhao H. Liang X. Li Y. (2023). Mesenchymal stem cells‐derived extracellular vesicles in orthopedic diseases: recent advances and therapeutic potential. Adv. Ther. 6, 2300193. 10.1002/adtp.202300193

[B35] Weber B. Sturm R. Henrich D. Marzi I. Leppik L. (2023). CD44+ and CD31+ extracellular vesicles (EVs) are significantly reduced in polytraumatized patients with hemorrhagic shock–evaluation of their diagnostic and prognostic potential. Front. Immunol. 14, 1196241. 10.3389/fimmu.2023.1196241 37662913 PMC10471799

[B36] Wiest E. F. Zubair A. C. (2020). Challenges of manufacturing mesenchymal stromal cell–derived extracellular vesicles in regenerative medicine. Cytotherapy 22, 606–612. 10.1016/j.jcyt.2020.04.040 32532592

[B37] Xu L. Liu Y. Sun Y. Wang B. Xiong Y. Lin W. (2017). Tissue source determines the differentiation potentials of mesenchymal stem cells: a comparative study of human mesenchymal stem cells from bone marrow and adipose tissue. Stem cell Res. and Ther. 8, 275–286. 10.1186/s13287-017-0716-x 29208029 PMC5718061

[B38] Yang Y.-H. K. Ogando C. R. Wang See C. Barabino G. A. (2018). Changes in phenotype and differentiation potential of human mesenchymal stem cells aging *in vitro* . Stem cell Res. and Ther. 9, 131–145. 10.1186/s13287-018-0876-3 29751774 PMC5948736

[B39] Yin B. Ni J. Witherel C. E. Yang M. Burdick J. A. Wen C. (2022). Harnessing tissue-derived extracellular vesicles for osteoarthritis theranostics. Theranostics 12, 207–231. 10.7150/thno.62708 34987642 PMC8690930

[B40] Yoon D. S. Kim Y. H. Lee S. Lee K. Park K. H. Jang Y. (2014). Interleukin‐6 induces the lineage commitment of bone marrow‐derived mesenchymal multipotent cells through down‐regulation of Sox2 by osteogenic transcription factors. FASEB J. 28, 3273–3286. 10.1096/fj.13-248567 24719354

[B41] Zhang J. Chen M. Liao J. Chang C. Liu Y. Padhiar A. A. (2021). Induced pluripotent stem cell-derived mesenchymal stem cells hold lower heterogeneity and great promise in biological research and clinical applications. Front. Cell Dev. Biol. 9, 716907. 10.3389/fcell.2021.716907 34660579 PMC8514743

