# Supplementary figures and images for: Batch variability and anti-inflammatory effects of iPSC-derived mesenchymal stromal cell extracellular vesicles in osteoarthritis in vitro model
Source: Front Bioeng Biotechnol. 2025 Apr 2;13:1536843. doi: 10.3389/fbioe.2025.1536843 (PMC11999995; doi:10.3389/fbioe.2025.1536843)

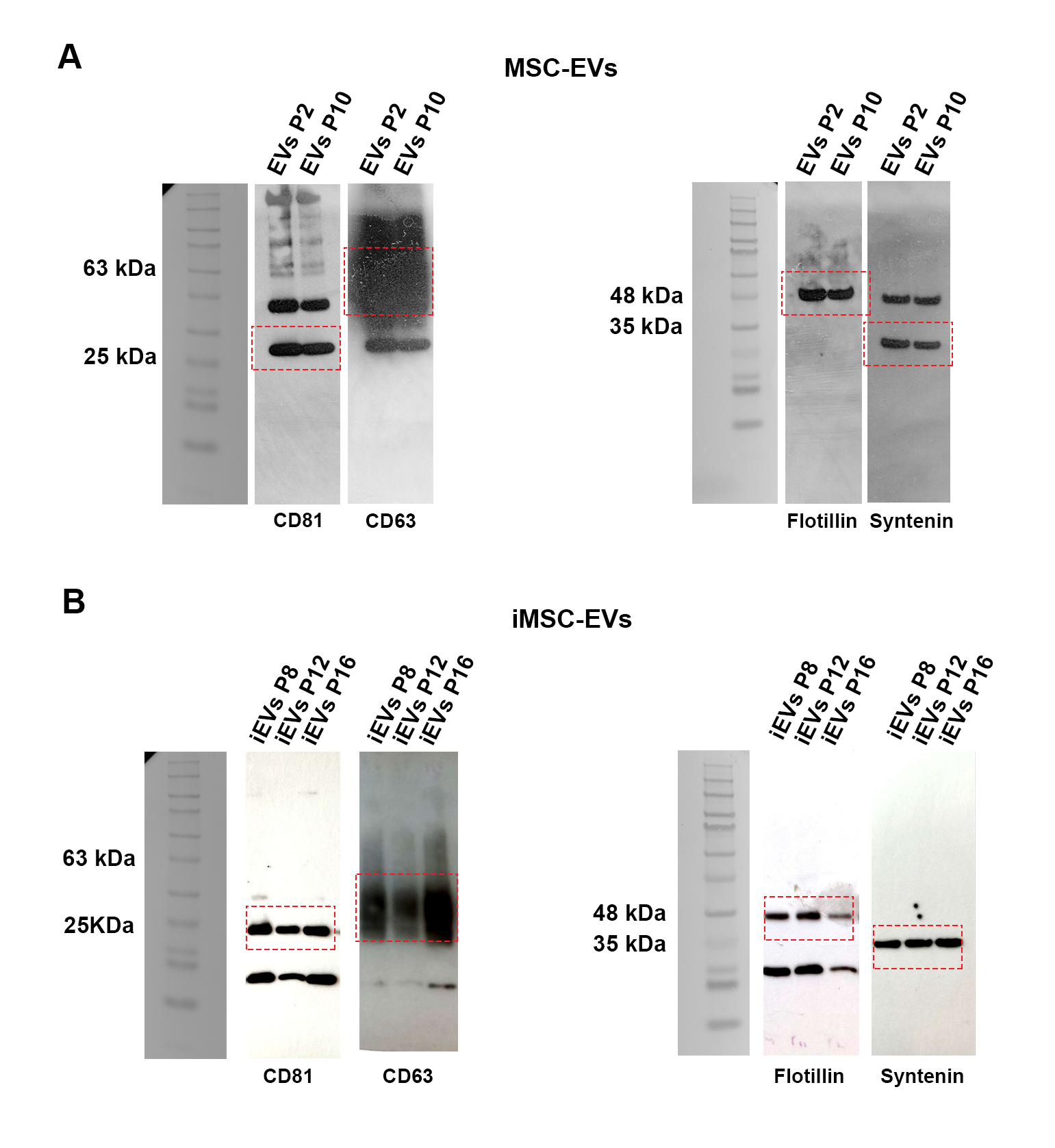

Supplement: Supplementary file 1 [file Image1.tif]
